# Fluorescent *Pseudomonas* Strains with only Few Plant-Beneficial Properties Are Favored in the Maize Rhizosphere

**DOI:** 10.3389/fpls.2016.01212

**Published:** 2016-08-25

**Authors:** Jordan Vacheron, Yvan Moënne-Loccoz, Audrey Dubost, Maximilien Gonçalves-Martins, Daniel Muller, Claire Prigent-Combaret

**Affiliations:** ^1^Université de LyonLyon, France; ^2^Université Lyon 1Villeurbanne, France; ^3^CNRS, UMR5557, Ecologie MicrobienneVilleurbanne, France; ^4^INRA, UMR1418Villeurbanne, France

**Keywords:** fluorescent *Pseudomonas*, PGPR, maize, functional groups, plant-beneficial properties

## Abstract

Plant Growth-Promoting Rhizobacteria (PGPR) enhance plant health and growth using a variety of traits. Effective PGPR strains typically exhibit multiple plant-beneficial properties, but whether they are better adapted to the rhizosphere than PGPR strains with fewer plant-beneficial properties is unknown. Here, we tested the hypothesis that strains with higher numbers of plant-beneficial properties would be preferentially selected by plant roots. To this end, the co-occurrence of 18 properties involved in enhanced plant nutrition, plant hormone modulation, or pathogen inhibition was analyzed by molecular and biochemical methods in a collection of maize rhizosphere and bulk soil isolates of fluorescent *Pseudomonas*. Twelve plant-beneficial properties were found among the 698 isolates. Contrarily to expectation, maize preferentially selected pseudomonads with low numbers of plant-beneficial properties (up to five). This selection was not due to the predominance of strains with specific assortments of these properties, or with specific taxonomic status. Therefore, the occurrence of only few plant-beneficial properties appeared favorable for root colonization by pseudomonads.

## Introduction

Plant roots interact with huge amounts of soil microorganisms, constituting their rhizomicrobiota, which participate to optimal plant development in response to environmental conditions. Among the rhizomicrobiota, certain bacterial strains, called Plant-Growth Promoting Rhizobacteria – PGPR, may exert beneficial effects on plant growth, development and/or health (Richardson et al., 2009; Vacheron et al., 2013). This entails various modes of action, related to improved nutrient supply (associative nitrogen fixation, phosphate solubilization, etc.), modulation of plant hormonal balance (*via* production of auxins, cytokinins, nitric oxide (NO), etc., or deamination of 1-aminocyclopropane-1-carboxylic acid (ACC)), and enhanced plant defense against parasites (through induction of systemic resistance pathways, or production of antimicrobial secondary metabolites, extracellular lytic enzymes, or surfactants; Spaepen et al., 2009; Vacheron et al., 2015).

It is not unusual that a given PGPR strain displays several different plant-beneficial properties (Loper et al., 2012; Bruto et al., 2014), which is thought to provide higher positive effects on the plant (Bashan and de-Bashan, 2010). This is expected to take place because (i) the effects of different modes of action may add-up quantitatively, or (ii) it could ensure that at least one mode of action is expressed in particular environmental conditions. Indeed, the most effective PGPR are typically multi-function strains (Rana et al., 2011; Almario et al., 2014a). In line with this, the loss of one mode of action often reduces plant-beneficial effects (Thomashow and Weller, 1988; Keel et al., 1990) while the gain of one may enhance plant-beneficial effects (Wang et al., 2000; Bakker et al., 2002; Holguin and Glick, 2003; Baudoin et al., 2010), and the combined use of PGPR strains with different modes of action can also improve the effects on the plant (Dunne et al., 1998; Combes-Meynet et al., 2011; Walker et al., 2012). Since strains with multiple plant-beneficial properties can provide higher benefits to the host, they might be more prevalent in the rhizosphere than related PGPR strains displaying a smaller number of plant-beneficial properties. This situation is reminiscent of the accumulation of virulence factors in pathogens, which allows a better fitness and host infection (Friesen et al., 2006). However, this hypothesis remains to be tested. Therefore, the objective of the present study was to assess whether the rhizosphere preferentially selects bacteria with high numbers of plant-beneficial properties.

To this end, we compared in various soils the effect of maize on the functional diversity of root-selected fluorescent *Pseudomonas* spp., a taxonomic group that displays a wide range of plant-beneficial properties and contains PGPR strains capable of phytostimulation and phytoprotection (Ahmad et al., 2008; Naik et al., 2008a,b; Loper et al., 2012; Rameshkumar et al., 2012; Sarma et al., 2014; Yadav et al., 2014). Plant-beneficial properties documented in these bacteria include associative nitrogen fixation (genes *nif*; Mirza et al., 2006), phosphate solubilization (Meyer et al., 2010), synthesis of the antimicrobial compound and root-branching signal 2,4-diacetylphloroglucinol (DAPG - genes *phl*; Keel et al., 1990; Leij et al., 2002; Brazelton et al., 2008), synthesis of the auxinic phytohormone indole-3-acetic acid (IAA; Oberhänsli et al., 1991) and the cytokinins isopentenyl adenosine and trans-zeatin ribose (García de Salamone et al., 2001), synthesis of the plant signal nitric oxide (nitrite reductase gene *nirS* or *nirK*; Arese et al., 2003; Baudouin and Hancock, 2014), and deamination of the plant’s ethylene precursor ACC (Glick et al., 1998; Prigent-Combaret et al., 2008). Synthesis of pyrrolnitrin, pyoluteorin (de Souza and Raaijmakers, 2003), phenazine (Mavrodi et al., 2006), hydrogen cyanide (HCN; Frapolli et al., 2012), and the production of extracellular lytic enzymes such as chitinases and proteases (Sarma et al., 2014) are also often reported as *Pseudomonas* plant-beneficial properties involved in biocontrol. Numerous fluorescent *Pseudomonas* strains were isolated from disease-conducive and -suppressive soils (including the MS8 soil used in the present study), and characterized merely according to the biocontrol properties they harbored (Ramette et al., 2003, 2006; Frapolli et al., 2008; Almario et al., 2014b). Here, a collection of fluorescent *Pseudomonas* isolates was established and screened for the presence of a wider range of well-described plant-beneficial properties, using established and novel methods targeting relevant genes, enzymatic activities and/or compounds. This isolate library was used to address the question whether fluorescent pseudomonads harboring a high number of co-occurring plant-beneficial properties are preferably selected in the maize rhizosphere.

## Materials and Methods

### Soils

Four soils were collected in October 2012 (Supplementary Table S1): MS8 from Morens, county Fribourg, Switzerland (46° 52′ 04.77″ N and 6° 54′ 15.83″ E; Kyselková et al., 2009), Bmo1 from Béligneux, Ain, France (45° 52′ 22.28″ N and 5° 7′ 53.21″ E), Ysa5 and Ysa8 from Seyssel, Savoie region, France (respectively, 45° 57′ 2.42″ N and 5° 51′ 11.44″ E; Almario et al., 2013), and 45° 58′ 30.42″ N and 5° 51′ 2.43″E). MS8 and Bmo1 have a morainic origin whereas Ysa5 and Ysa8 developed on sandstone material. Bmo1 and Ysa5 were cultivated with maize for at least 3 years, MS8 was an artificial meadow for at least 2 years, and Ysa8 is a natural grassland. Soils were taken from 10 to 30 cm depth at three locations 5–10 m apart in each field, and were sieved at 0.5 mm.

### Plant Experiment

Seeds of hybrid maize cultivar DK315 (Monsanto SAS/Dekalb, USA) and PR37Y15 (Pioneer Semences SAS, France) were surface-sterilized by soaking 1 h in sodium hypochlorite and one wash in 70% ethanol, and were rinsed three times with sterile distilled water. Three seeds of each maize variety were sown each in 2-dm^3^ pots (2 kg fresh soil/pot; 3 pots/cultivar/soil) and soil water content was maintained at 20% w/w. Unplanted pots (1 pot/soil) served as controls for sampling bulk soils. 21 days after sowing, rhizosphere soils and bulk soils were sampled (i.e., 3 root-adhering soils × 2 cultivars × 4 soils and 4 bulk soils). The root systems were shaken vigorously and rhizosphere extracts were prepared by putting each root system with adhering soil in 20 mL of 0.9% NaCl solution and shaking 1 h at 150 rpm. Four bulk soil extracts were obtained using 10 g soil (in 20 mL).

### Isolation and Characterization of *Pseudomonas* Isolates

For isolation of fluorescent *Pseudomonas*, the 24 rhizosphere and four bulk soil extracts were serially diluted and 20 μL was mixed with 180 μL of King’s B^+++^ [i.e., King’s B supplemented with ampicillin (40 mg/mL), chloramphenicol (13 mg/mL); Simon and Ridge, 1974; Landa et al., 2002; Latz et al., 2016] in 96-well microtiter plate following a most probable number (MPN) design with eight wells per dilution. Aliquots from each last positive well were plated on King’s B^+++^ agar. At least 55 isolates were randomly selected for each of the 12 conditions [i.e., 4 soils × (2 cultivars and bulk soil)] and all colonies were purified three times successively, giving a total of 698 isolates. Genomic DNA was extracted for all isolates using NucleoSpin^®^ 96 Tissue kits (Ref - 740454.4; Macherey Nagel, Germany) and identification performed by sequencing the housekeeping gene *rpoD* (accession numbers LN885567 to LN886065, EMBL-EBI database) using primers rpoDf/rpoDr targeting the *rpoD* alleles of bacteria from the *P. fluorescens* group (Frapolli et al., 2007). When *rpoD* amplification failed, the 16SrRNA encoding *rrs* gene was amplified with pA/pH (Edwards et al., 1989) and sequenced (accession numbers: LN885368 to LN885566, EMBL-EBI database). *rpoD* sequences were aligned with MUSCLE (Edgar, 2004). Sequences were manually filtered to discard gaps and aligned regions of low quality. The phylogenetic trees were inferred with PHYML (Guindon et al., 2010) with the GTR model and 500 bootstraps. Isolate redundancy was estimated at 30%, according to both *rpoD* sequence similarity and functional profiles obtained.

### Screening of *Pseudomonas* Isolates for Plant-Beneficial Properties

A total of 18 plant-beneficial properties were targeted. Molecular screening for plant-beneficial properties was performed by PCR targeting genes involved in production of 2,4 diacetylphloroglucinol (*phlD*), pyrrolnitrin (*prnD*), pyoluteorin (*pltC*), phenazines (overlapping region of *phzC*/*phzD*) and NO (*nir*S in pseudomonads), as well as ACC deamination (*acdS*) and nitrogen fixation (*nifH*). All amplifications were performed with a thermocycler Mastercycler (Eppendorf, Germany). The reaction volumes contained 10x PCR buffer, 50 mM MgCl_2_, 2 mM dNTP, 5% DMSO, 10 μM of each primer (Supplementary Table S2), 1 unit of Taq polymerase (Invitrogen, Cergy-Pontoise, France) and 50 ng of DNA. Several amplified fragments were sequenced and data blasted against the NCBI database in order to ascertain that isolates actually harbored the corresponding genes (accession numbers LT607759 to LT607801, EMBL-EBI database). No false-positive PCR results were found.

Screening for isolates with phosphate solubilizing activity was done by measuring the degradation halo on a National Botanical Research Institute’s Phosphate (NBRIP) agar after 6 days at 28°C, according to (Meyer et al., 2011). HCN production (indicated by color orange to red) was assessed after growth (3 days at 28°C) on King’s B^+++^ agar, using a Whatman filter paper n°1 previously soaked in 2% sodium carbonate in 0.5% picric acid solution and placed in the lid of the Petri dish (subsequently sealed with parafilm). Production of extracellular protease and chitinase was assessed using, respectively, milk agar and minimum medium supplemented with colloidal chitin (Kim et al., 2003).

An Ultra High Performance Liquid Chromatography (UHPLC) method was developed in order to screen isolates for production of the two auxinic phytohormones indole-3-acetic acid (IAA) and indol-3-butyric acid and the five cytokinin phytohormones *trans*-zeatin, *trans*-zeatin riboside, kinetin, 6-benzylaminopurine and isopentenyl adenosine. Briefly, all isolates were grown in 2 mL of King’s B medium supplemented with 250 mM of auxin precursor tryptophan and 0.1 mM of cytokinin precursor adenine (2 days at 28°C, 300 rpm). The cultures were centrifuged at 4500 rpm during 8 min and filtered at 0.2 μm. Supernatants were subjected to UHPLC separation on an Agilent 1290 Series instrument using a 100 mm × 3 mm reverse phase column (Agilent Poroshell 120 EC-C18, 2.7 μm particle size) with a diode array detector. Samples (10 μL) were loaded onto the column equilibrated with water and acetonitrile (98:2). Compounds were eluted by a two-step gradient increasing the acetonitrile concentration to 40% over a 6 min period, then to 100% over 4 min, followed by an isocratic step of 2 min, at a flow rate of 0.5 mL/min. Hormones were detected with an Agilent 6530 Quadrupole Time-of-Flight (Q-TOF) mass spectrometer in positive electrospray ionization, based on comparison with commercial standards on both mass and UV (280 nm) chromatograms, along with accurate mass and UV spectra.

### Statistical Analysis

Heatmaps were analyzed using R “pheatmap” package (Kolde, 2012). Clustering analysis was performed using Euclidean distance method or Spearman correlation. For each condition, data were log-transformed for normal distribution and variance homogeneity, and a two-way ANOVA and Tukey’s HSD tests were performed to detect soil or variety impact on fluorescent *Pseudomonas* population size. *Pseudomonas* proportions were compared with a χ^2^ test, or Fisher’s exact test when the expected values in any of the cells of a contingency table were below 5. Numbers of *Pseudomonas* isolates were compared by ANOVA and Fisher’s LSD tests. All analyses were performed at *P* < 0.05, using R software (Venables and Smith, 2011). Results in text and figures are presented as means ± standard error.

## Results

### Enumeration of Culturable Fluorescent *Pseudomonas* in Bulk Soil and Maize Rhizospheres

In all conditions, culturable fluorescent *Pseudomonas* populations were significantly more abundant in rhizosphere soil than in the corresponding bulk soil (Supplementary Figure S1). The mean number of culturable fluorescent *Pseudomonas* did not differ according to the maize cultivar (7.09 ± 0.26 log cells/g for PR37Y15 and 6.99 ± 0.22 log cells/g for DK315; *P* = 0.064), but the interaction between soils and cultivars was significant (*P* = 0.032). Indeed, in Ysa5 soil, pseudomonads were more abundant in PR37Y15 than in DK315 maize rhizosphere, whereas no significant differences were observed between cultivars in the three other soils. In addition, the number of culturable fluorescent pseudomonads in the rhizosphere differed according to the soil for cultivar PR37Y15 but not for DK315 (Supplementary Figure S1).

### Distribution of Plant-Beneficial Properties in Bulk Soils and Rhizospheres

**FIGURE 1:**
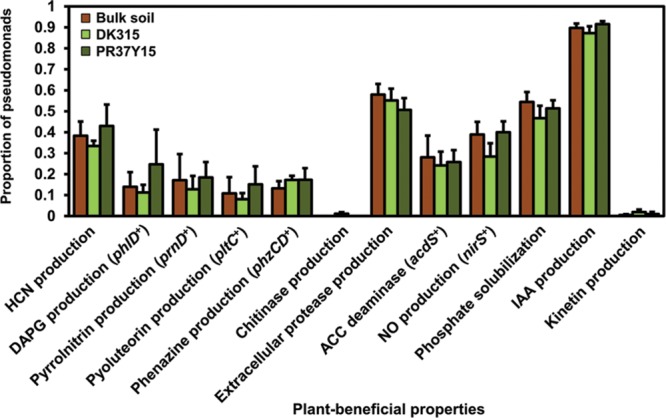
**Proportion of pseudomonads according to the type of plant-beneficial properties harbored, in bulk soil and in DK315 and PR37Y15 maize rhizospheres.** Data from soils Bmo1, Ysa5, MS8, and Ysa8 were combined and error bars (standard errors) reflect the variability among the four soils. HCN, hydrogen cyanide; DAPG, 2,4-diacetylphloroglucinol; ACC, 1-amino-cyclopropane carboxylic acid; NO, nitric oxide.

**FIGURE 2:**
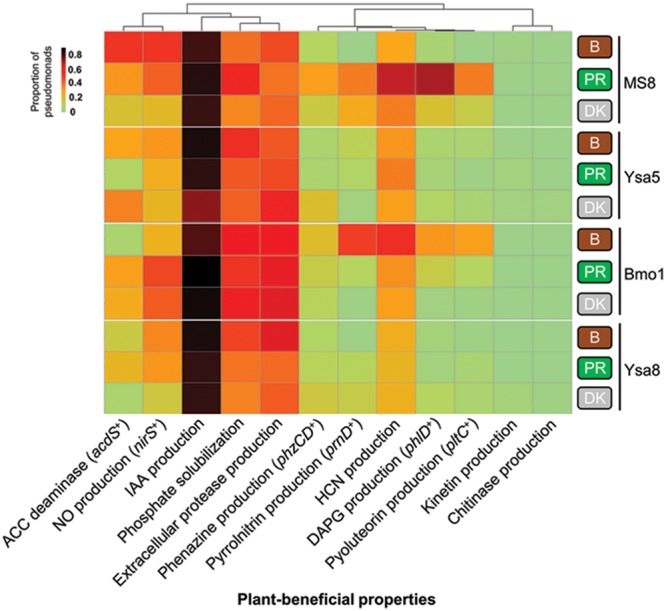
**Prevalence of plant-beneficial properties in pseudomonads in bulk soil and in DK315 and PR37Y15 maize rhizospheres at 21 days in soils Bmo1, Ysa5, MS8, and Ysa8.** The heatmap represents the proportion of fluorescent pseudomonads harboring each plant-beneficial property amongst the 12 targeted and detected ones. Column clustering was performed using the correlation method. B, Bulk soil (brown rounded rectangles); PR, cultivar PR37Y15 (green rounded rectangles); DK, cultivar DK315 (gray rounded rectangles).

Among the 698 isolated *Pseudomonas*, 209 isolates were isolated from PR37Y15 maize rhizospheres, 255 from DK315 rhizospheres and 234 from bulk soils (**Table 1**). A total of 18 plant-beneficial properties were targeted among the 698 *Pseudomonas* isolates obtained. Whereas the nitrogen fixation gene *nifH*, as well as the ability to synthesize indole-butyric acid, *trans*-zeatin, *trans*-zeatin riboside, isopentenyl adenosine or 6-benzylaminopurine, were not found in any isolates from the library, the 12 other plant-beneficial properties studied were found in 2% (extracellular chitinase activity) to 96% (production of IAA) of the 698 pseudomonads. When considering together the 12 displayed plant-beneficial properties, a total of 175 different combinations (i.e., functional profiles) were found among the 698 isolates. When data from all soils were pooled together, no significant differences were observed when comparing the proportion of isolates displaying a given plant-beneficial property, in bulk soil and PR37Y15 and DK315 maize rhizospheres, regardless of the property tested (χ^2^ tests, *P* > 0.05 for each plant-beneficial property; **Figure 1**). When soil treatments were analyzed separately (**Figure 2**), the distribution of these properties in pseudomonads differed according to soil type and maize cultivar, especially for properties involved in the biosynthesis of antimicrobial compounds (i.e., DAPG, pyrrolnitrin, pyoluteorin) found in lower proportion in PR37Y15 and DK315 maize rhizospheres than bulk soil for Bmo1 (*P-*value of χ^2^ tests from 10^-10^ to 0.04). For the same soil, however, the proportion of *Pseudomonas* harboring *acdS* gene was higher in both maize rhizospheres than bulk soil (*P* < 0.01; **Figure 2**). Conversely, in MS8 soil, proportions of *Pseudomonas* harboring genes involved in the biosynthesis of antimicrobial compounds (i.e., DAPG, pyrrolnitrin, pyoluteorin and phenazine) were higher in both maize rhizospheres than bulk soil (*P-*value of χ^2^ tests from 10^-12^ to 0.05) whereas *acdS* was higher in bulk soil than both maize rhizospheres (*P* < 0.05). In the other two soils, no significant differences were detected for the distribution of each property.

**Table 1:** Taxonomical distribution of isolates among fluorescent *Pseudomonas* groups and subgroups according to their origin of isolation.

Groups	Subgroups	MS8^†^			Ysa5			Ysa8			Bmo1			Total
		Bulk soil	PR37Y15	DK315	Bulk soil	PR37Y15	DK315	Bulk soil	PR37Y15	DK315	Bulk soil	PR37Y15	DK315	
*P. fluorescens* (cluster FMJK)	*P. fluorescens*	1	19	17	2	12	8	–	18	11	1	15	37	141
	*P. mandelii*	21	3	12	4	–	–	1	–	2	4	–	1	48
	*P. jessenii*	6	10	6	6	2	1	22	1	2	7	11	6	80
	*P. koreensis*	11	2	13	4	4	12	–	7	23	2	7	13	98
*P. fluorescens* (cluster CPC)	*P. corrugata*	–	1	–	1	–	1	–	1	1	19	1	–	25
	*P. chlororaphis*	–	–	–	10	4	4	11	5	7	–	–	–	41
	*P. protegens*	–	4	–	6	1	–	24	2	–	26	1	1	65
Total *P. fluorescens*		39	39	48	33	23	26	58	34	46	59	35	58	498
*P. putida*		15	18	10	27	27	43	2	23	16	1	10	8	200
	**Total**	54	57	58	60	50	69	60	57	62	60	45	66	698

*^†^Physical and chemical characteristics of soils used in this study are given in Supplementary Table S1.*

When considering the numbers (rather than relative proportions) of fluorescent *Pseudomonas* – estimated by multiplying the *Pseudomonas* proportions with the raw population levels from each bulk and rhizosphere soil, and thereafter pooling together data from all soils – a higher number of pseudomonads harboring each property studied was found in the rhizosphere of one (i.e., PR37Y15 for *phlD, prnD, pltC*) or both maize cultivars (i.e., *phzCD, acdS, nirS*, extracellular protease activity, HCN and IAA production, phosphate solubilization) than in the bulk soil, by approximately two orders of magnitude (Supplementary Figure S2). Numbers of kinetin producers and of pseudomonads with chitinase activity were below 2 log cells/g in all three treatments.

### Distribution of *Pseudomonas* Isolates in Bulk Soils and Rhizospheres According to the Number and Assortment of Plant-Beneficial Properties They Harbor

**FIGURE 3:**
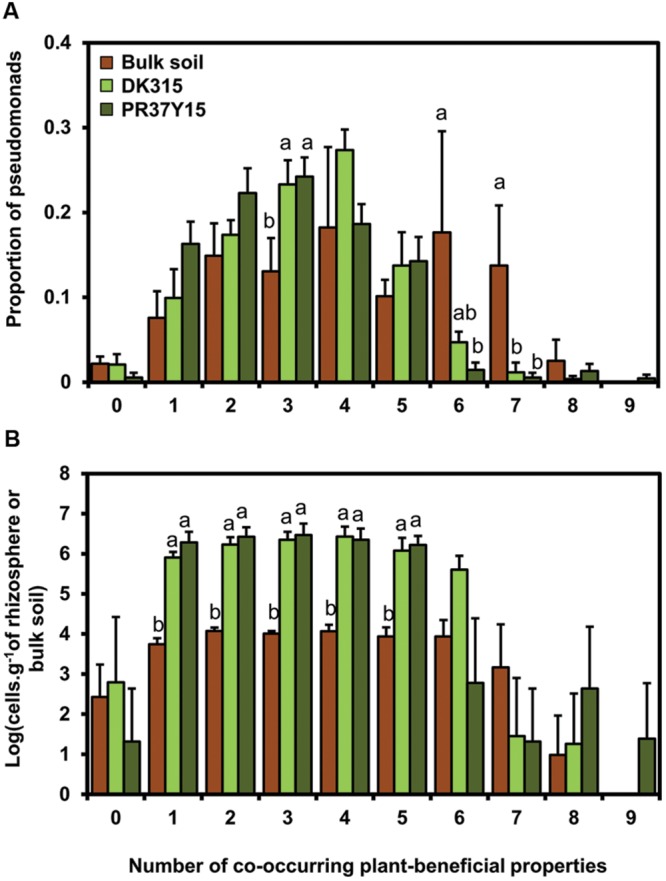
**Proportion **(A)** and estimated number **(B)** of pseudomonads according to the number of co-occurring plant-beneficial properties harbored (from 0 to 9), in bulk soil and in DK315 and PR37Y15 maize rhizospheres.** Data from soils Bmo1, Ysa5, MS8, and Ysa8 were combined and error bars (standard errors) reflect the variability among the four soils. The number of *Pseudomonas* was estimated by multiplying the *Pseudomonas* proportions with the population levels quantified in each bulk and rhizosphere soil. Statistical differences between conditions (bulk soil/PR37Y15/DK315) are indicated with letter a-b (ANOVA and Fisher’s LSD tests. *P* < 0.05).

*Pseudomonas* isolates possessed from 0 (11 isolates) to a maximum of 9 (1 isolate) co-occurring plant-beneficial properties (i.e., functions harbored by a same isolate) among the 18 plant-beneficial properties targeted, with a mean of 3.6 properties per isolate. To assess whether maize preferentially selects fluorescent *Pseudomonas* harboring a high number of co-occurring plant-beneficial properties, the number of co-occurring plant-beneficial properties per isolate was analyzed according to their origin of isolation (rhizospheres or bulk soils). When data from all soils were pooled together, most rhizosphere isolates (433 of 464 rhizosphere isolates, i.e., 93%) displayed one to five co-occurring plant-beneficial properties per isolate, whereas 81 of 234 bulk soil isolates (i.e., 35%) displayed six to nine of them (**Figure 3A**). When considering each soil separately, the same proportion distribution of *Pseudomonas* sharing 0 to 9 co-occurring plant-beneficial properties per isolate was found in the maize PR37Y15 and DK315 rhizospheres (from *P* = 0.32 for Ysa8 soil to *P* = 0.56 for Bmo1 soil; Supplementary Figure S3). In addition, the distribution was similar for bulk soil and rhizosphere in MS8 and Ysa5 soils (Supplementary Figures S3A,B) whereas it differed in soils Ysa8 and Bmo1 (Supplementary Figures S3C,D). When population levels were estimated for fluorescent *Pseudomonas* harboring various numbers of co-occurring plant-beneficial properties and data from all soils were pooled together, it appeared that the numbers of culturable fluorescent *Pseudomonas* harboring up to five plant-beneficial properties reached about 6 log cells/g in the maize rhizosphere of both PR37Y15 and DK315, but only 4 log cells/g in bulk soil (*P* < 0.001; **Figure 3B**). In contrast, pseudomonads with six to nine plant-beneficial properties were found in the maize rhizospheres in similar substantial numbers, with 2.25 ± 0.47 log cells/g for PR37Y15 and 2.77 ± 1.42 log cells/g for DK315 compared with bulk soil (2.70 ± 0.88 log cells/g; **Figure 3**).

Among the 698 isolates, the ratio of the number of functional profiles (i.e., different associations of plant-beneficial properties in isolates) to the number of isolates was significantly higher (*P* = 0.030) in PR37Y15 (0.56 ± 0.02) compared to DK315 (0.45 ± 0.03) maize rhizosphere and bulk soil (0.45 ± 0.05). However, correspondence analysis indicated that bulk soil and rhizospheres overlapped completely when assessing *Pseudomonas* isolate profiles (Supplementary Figure S4). Results thus point to a lack of selection of pseudomonads with specific assortments of plant-beneficial properties in the rhizosphere.

Some plant-beneficial properties preferentially co-occurred in *Pseudomonas* isolates. Genes for synthesis of organic antimicrobial compounds (i.e., DAPG, pyrrolnitrin, pyoluteorin and phenazines) grouped together following hierarchical clustering analysis (**Figure 2**) and significantly correlated with one another (Supplementary Figure S5). Likewise, properties involved in modulation of plant hormonal balance (i.e., ACC deaminase activity gene *acdS* and NO production gene *nirS*, which correlated positively; *r* = 0.70, *P* < 0.011, Supplementary Figure S5), phosphate solubilization, extracellular protease activity and HCN production grouped together (**Figure 2**).

### Taxonomic Characterization of Fluorescent *Pseudomonas* Isolates

**FIGURE 4:**
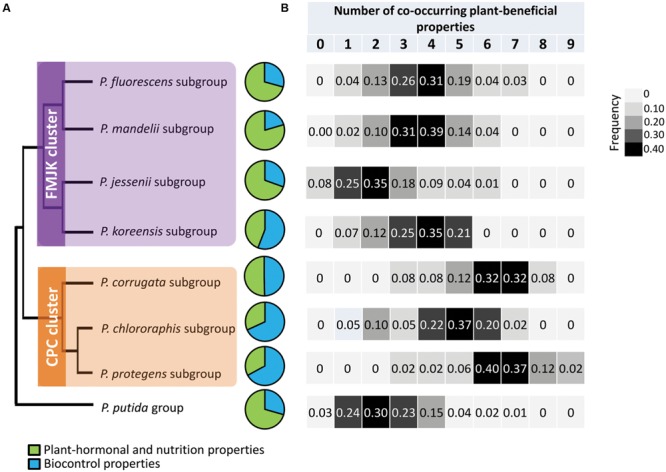
**Distribution of plant-beneficial properties in *Pseudomonas* isolates according to **(A)** their taxonomic affiliation and **(B)** number of co-occurring plant-beneficial properties per isolate among the 18 plant-beneficial properties analyzed.** The topology of the cladogram has been drawn according to the phylogenetic classification of Gomila et al. (2015). Both clusters are indicated. Biocontrol properties include production of antimicrobial compounds such as DAPG, pyrrolnitrin, pyoluteorin, phenazine, and HCN, and production of extracellular protease and chitinase. Plant hormonal and nutrition properties regroup ACC deaminase, production of NO, kinetin and IAA and phosphate solubilization.

In order to characterize taxonomically the *Pseudomonas* isolates, *rpoD* amplification and sequencing were performed using specific primers for the *P. fluorescens* group, which was successful for 498 isolates (60% of PR37Y15 isolates, 82% for DK315 isolates and 81% of bulk soil isolates; i.e., 70% overall *Pseudomonas* isolates). The 16S rRNA *rrs* gene sequencing on the 200 remaining isolates affiliated all of them to the *P. putida* group. *rpoD* phylogeny (Supplementary Figure S6) enabled affiliation of the *rpoD*-sequenced isolates to 7 of the 9 subgroups of the *P. fluorescens* group proposed by Garrido-Sanz et al. (2016), i.e., the subgroups *P. corrugata* (3.6% of isolates), *P. chlororaphis* (5.9%), *P. protegens* (9.3%), *P. fluorescens* (20.2%), *P. mandelii* (6.9%), *P. jessenii* (11.5%), or *P. koreensis* (14.0%; **Figure 4A**; Supplementary Figure S6; **Table 1**). According to the phylogenies of Gomila et al. (2015) and Garrido-Sanz et al. (2016), two clusters, hereafter termed clusters FMJK (for the *P. fluorescens, P. mandelii, P. jessenii, P. koreensis* subgroups) and CPC (for the *P. chlororaphis, P. protegens, P. corrugata* subgroups), were defined in this study.

### Distribution of Plant-Beneficial Properties According to *Pseudomonas* Subgroups

The distribution of plant-beneficial properties was assessed according to their affiliation to the 7 *P. fluorescens* subgroups recovered in this study (**Figure 4**; **Table 2**). The *P. chlororaphis* subgroup shared the highest functional diversity with 12 distinct plant-beneficial properties recovered among isolates from this subgroup (regardless of their co-occurrence) whereas strains affiliated to *P. koreensis* subgroup shared the lowest functional diversity (i.e., 8 distinct plant-beneficial properties; **Table 2**). The number of plant-beneficial properties found per isolate (i.e., co-occurring properties) was significantly lower (*P* < 0.001) for the FMJK cluster (3.36 ± 0.07 properties per isolate) than the CPC cluster (5.76 ± 0.13 properties; **Figure 4B**).

**Table 2:** Distribution of plant-beneficial properties among fluorescent pseudomonads according to taxonomical groups and subgroups they belong to.

Groups	Subgroups	HCN^+^	*phlD^+^*^†^	*prnD^+^*	*pltC^+^*	*phzCD^+^*	Chitinase ^+^	Protease ^+^	IAA^+^	Kinetin^+^	*acdS^+^*	*nirS^+^*	Phosphate solubilization^+^
*P. fluorescens* (cluster FMJK)	*P. fluorescens*	0.04	0.06	0.06	0.05	0.04	0.01	0.83	0.9	0	0.38	0.59	0.77
	*P. mandelii*	0.18	0.12	0	0	0.08	0	0.37	0.86	0	0.69	0.88	0.47
	*P. jessenii*	0.17	0	0.1	0.04	0.05	0	0.23	0.7	0	0.18	0.21	0.39
	*P. koreensis*	0.84	0	0	0	0.41	0	0.8	0.9	0.02	0.04	0.13	0.52
*P. fluorescens* (cluster CPC)	*P. corrugata*	0.64	0.76	0.12	0	0.64	0	0.8	1	0.04	0.88	0.92	0.16
	*P. chlororaphis*	0.73	0.07	0.59	0.05	0.76	0.02	0.8	0.83	0.02	0.05	0.15	0.37
	*P. protegens*	0.91	0.97	0.98	0.98	0	0	0.52	0.98	0	0.02	0.17	0.98
*P. putida* group		0.26	0	0.03	0.02	0.06	0	0.32	0.94	0.02	0.25	0.25	0.3

*Number correspond to the proportion of isolates harboring plant-beneficial properties according to their affiliation to the taxonomic groups and subgroups. ^†^*phlD, prnD, pltC, phzCD, acdS*, and *nirS*, correspond to key genetic determinants involved, respectively, in DAPG, pyrrolnitrin, pyoluteorin, phenazine, ACC deaminase and nitric oxide productions.*

The ecological functions conferred by plant-beneficial properties harbored by isolates also differed according to the taxonomic affiliation of *Pseudomonas* isolates. When regrouping plant-beneficial properties according to their type of action, differences between *Pseudomonas* subgroups were observed regarding the proportion of (i) biocontrol properties (i.e., genes for antimicrobial compounds DAPG, pyrrolnitrin, pyoluteorin and phenazine, and production of extracellular protease, chitinase, and HCN) and (ii) properties related to plant hormonal balance modulation and plant nutrition (i.e., ACC deaminase and NO genes, production of kinetin and IAA, and phosphate solubilization; **Figure 4A**). Overall, higher proportions of biocontrol properties were found in isolates belonging to the CPC cluster (68% for *P. chlororaphis*, 67% for *P. protegens* and 50% for *P. corrugata* subgroups) than among most members of the FMJK cluster (29% for *P. fluorescens*, 20% for *P. mandelii*, 30% for *P. jessenii* and 56% for *P. koreensis* subgroups; **Figure 4A**). Conversely, phytostimulatory properties (i.e., plant hormone modulation and nutrition properties) were found, for the most part, in higher proportion in FMJK isolates (71% for *P. fluorescens*, 80% for *P. mandelii*, 70% for *P. jessenii* and 44% for *P. koreensis* subgroups) than CPC isolates (32% for *P. chlororaphis*, 33% for *P. protegens* and 50% for *P. corrugata* subgroups; **Figure 4A**).

The proportions of all individual biocontrol properties in the pseudomonads were also higher (*P* < 0.001 each) in cluster CPC than FMJK (**Table 2**). Contrariwise, all properties related to plant hormonal balance modulation and plant nutrition were found in similar proportions in CPC and FMJK clusters.

Isolates belonging to the *P. putida* group displayed more plant-beneficial properties related to plant hormonal balance modulation and plant nutrition than to biocontrol (**Figure 4A**). Moreover, these isolates harbored a low number of co-occurring plant-beneficial properties, i.e., 2.5 ± 0.1 (**Figure 4B**).

### Maize Selection and Distribution of *Pseudomonas* CPC and FMJK Clusters and of *P. putida*

**FIGURE 5:**
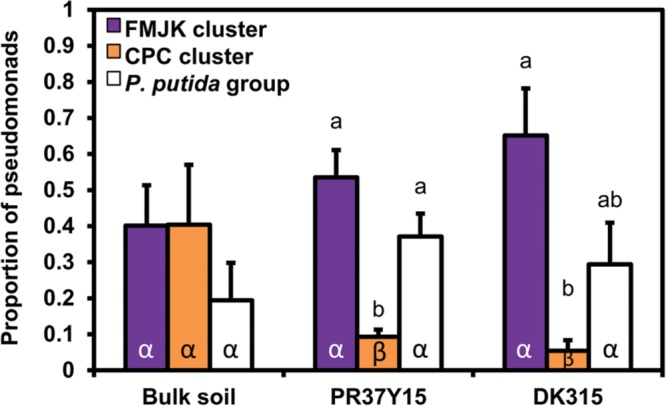
**Comparison of the proportion of pseudomonads belonging to FMJK and CPC clusters and to *P. putida* group in bulk soil and in the PR37Y15 and DK315 maize rhizospheres.** Error bars correspond to standard errors. Statistical differences between subgroups are indicated with letter a-b and statistical differences between conditions (bulk soil/PR37Y15/ DK315) are indicated with letter α-β (ANOVA and Fisher’s LSD tests. *P* < 0.05).

The FMJK and CPC clusters were present in the same relative proportion (40%) in bulk soil, but in the rhizosphere, FMJK isolates were retrieved in a significantly higher proportion than CPC isolates (up to 60% for FMJK versus below 10% for CPC; **Figure 5**). The *P. putida* group was found in the same proportion in bulk soil and in the rhizosphere. The proportion of this group was significantly more important than that of the CPC cluster in PR37Y15 maize rhizosphere (**Figure 5**). The same trends were observed when considering the estimated number of *Pseudomonas* from FMJK and CPC clusters, in bulk soils or rhizospheres (Supplementary Figure S7).

**FIGURE 6:**
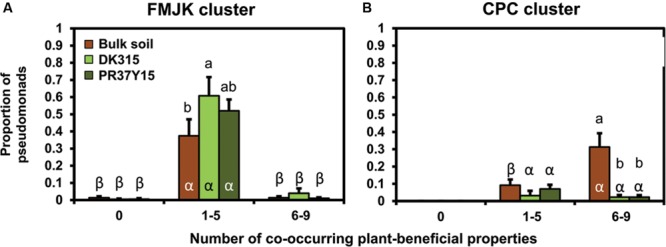
**Comparison of the proportion of pseudomonads belonging to FMJK **(A)** and CPC **(B)** clusters in bulk soil and in the PR37Y15 and DK315 maize rhizospheres according to the number of co-occurring plant-beneficial properties harbored.** Error bars correspond to standard errors. Statistical differences between plant-beneficial property classes according to conditions (bulk soil/PR37Y15/DK315) are indicated with letter α-β (ANOVA and Fisher’s LSD tests. *P* < 0.05). Statistical differences between conditions within plant-beneficial property class are indicated with letter a-b.

When considering the amounts of co-occurring plant-beneficial properties per isolate, FMJK isolates with one to five plant-beneficial properties were more abundant than FMJK isolates with six to nine plant-beneficial properties in all soils (*P* < 0.001; **Figure 6A**). This was also the case when considering estimated *Pseudomonas* population levels (Supplementary Figure S7B).

In parallel, the proportions of isolates belonging to CPC cluster with six to nine plant-beneficial properties were smaller in the rhizospheres of both cultivars (*P* = 0.002) than in bulk soil, whereas those with one to five plant-beneficial properties were found in similar proportions in bulk soils and maize rhizospheres (*P* = 0.28; **Figure 6B**). When considering estimated *Pseudomonas* population levels, the numbers of CPC isolates with one to five or six to nine plant-beneficial properties were similar between bulk soil and maize rhizospheres, except in the PR37Y15 maize rhizosphere where a higher number of CPC isolates with one to five plant-beneficial properties was found than in the other conditions (Supplementary Figure S7C).

In conclusion, the selection of *Pseudomonas* strains in the maize rhizosphere depends more on the number of plant-beneficial properties they harbor than the *Pseudomonas* cluster they belong to.

## Discussion

The fluorescent *Pseudomonas* are key models to assess beneficial plant–bacteria interactions, because they display a wide range of plant-beneficial properties and play an important role in the rhizosphere, including in disease-suppressive soils (Weller and Cook, 1983; Lemanceau and Alabouvette, 1991; O’Sullivan and O’Gara, 1992; Bakker et al., 2007; Barret et al., 2009; Almario et al., 2014b). A given *Pseudomonas* PGPR strain generally displays many different modes of action on the plant, which is thought to be important to maximize plant benefits. However, contrarily to expectations, we found that *Pseudomonas* rhizobacteria with high numbers (>5) of plant-beneficial properties were not prevalent in the maize rhizosphere in our experimental conditions, as they were outcompeted by counterparts with lower numbers (1–5) of these properties. Therefore, even though *Pseudomonas* inoculants with high numbers of plant-beneficial properties are being sought in PGPR screening programs (Ahmad et al., 2008), their high effectiveness in short-term greenhouse trials may be counterbalanced by insufficient rhizosphere survival under field conditions, an issue long identified (Weller, 1988).

The current finding is hard to track back to literature data, as direct comparisons of *Pseudomonas* strains with 1–5 vs. 6–9 plant-beneficial properties are not available. The high-property-number CPC-cluster strain *P. protegens* CHA0 survived poorly in the rhizosphere of well-established maize and wheat (Troxler et al., 1997) and was seldom evidenced by *phlD* PCR-DGGE even in its habitat of origin, i.e., roots of tobacco grown in Morens suppressive soils (Frapolli et al., 2010). In contrast, pseudomonads belonging to the FMJK cluster that overall possess fewer plant-beneficial properties were prominent in, for instance, the strawberry rhizosphere based on *rrs* PCR-DGGE (Costa et al., 2006) and *gacA* PCR-DGGE (Costa et al., 2007), whereas pseudomonads related to *P. protegens* CHA0 were not found.

Eighteen contrasted plant-beneficial properties were screened in the *Pseudomonas* collection and 12 were evidenced. We recognize that results of the present study depended on the number of tested properties. Other properties like the biosynthesis of flagella, exopolysaccharides, lipopolysaccharides, or siderophores are pivotal for the colonization of host plants and adaptation to the rhizosphere (Höfte et al., 1992; Duijff et al., 1997; de Weert et al., 2002; Barahona et al., 2011). These properties are widely distributed in *Pseudomonas* and may not be sufficiently discriminant to use them for characterizing *Pseudomonas* isolate library (Luján et al., 2015).

In the maize rhizosphere, auxin production is widespread in fluorescent *Pseudomonas* (Picard et al., 2004; Picard and Bosco, 2005), as was confirmed here, but (i) it is often deduced from the Salkowski colorimetric assay, which responds also to other indolic compounds (Glickmann and Dessaux, 1995; Szkop and Bielawski, 2012), and (ii) different auxins may play different roles in plant (Simon and Petrášek, 2011). Therefore, a UHPLC approach coupled with a diode array detector was implemented to detect different types of auxins (and cytokinins), which showed that only indole-3-acetic acid (and the cytokinin kinetin) was produced in pure culture by pseudomonads. We cannot exclude that the other types of tracked auxins and cytokinins could be produced by *Pseudomonas* isolates in other conditions, such when interacting with the host plant. The gene *nifH*, which occurs in certain *Pseudomonas* strains (Mirza et al., 2006), was not evidenced here.

The distribution of plant-beneficial properties in pseudomonads was not random, as co-occurrence patterns were found. This concerned *acdS* (ACC deaminase) and *nirS* (nitrite reductase), both involved in the modulation of plant hormonal balance and plant defenses (Glick, 2005; Vacheron et al., 2015), which suggests possible fine tuning of plant hormonal conditions by rhizosphere *Pseudomonas* populations. It was also the case for *acdS* and indole-3-acetic acid, in line with the observation that auxins stimulate ACC synthase activity in roots (Glick et al., 1998). Finally, the co-occurrence of genes for production of organic antimicrobial compounds, which is well documented in model strains (Ramette et al., 2003; Haas and Défago, 2005; Raaijmakers et al., 2006; Mazurier et al., 2009), raises the possibility of additive or even synergistic effects in phytoprotection (Clifford et al., 2015).

A broader study analyzed the distribution of plant-beneficial properties among a total of 304 genomes of Proteobacteria with different ecologies (Bruto et al., 2014). Among the 25 PGPR sequenced genomes, the co-occurrence between *phl* and *hcn* genes was found. In contrast, no relation was described between the ACC deaminase gene and the nitrite reductase gene involved in NO production (Bruto et al., 2014), a co-occurence that we observed in the present study. This can be explained by the fact that the present study targeted a lower taxonomic level (i.e., *Pseudomonas fluorescens* group and *Pseudomonas putida* group). In addition, the identification of specific co-occurrence between plant-beneficial properties may be influenced by phylogenetic signal phenomenon, i.e., the tendency of related species to be more similar to each other than two species taken randomly from a same phylogenetic tree (Münkemüller et al., 2012; Bruto et al., 2014).

Differences in plant-beneficial property distributions were found between (i) the four soils (**Figure 2**), (ii) bulk soil and rhizosphere, and (iii) the two maize cultivars, as could be expected in relation to documented rhizosphere effects (Marschner et al., 2001; Berg and Smalla, 2009; İnceoǧlu et al., 2012). In the current study, the plant-beneficial profiles harbored by pseudomonads in DK315 rhizosphere were less diversified than those found in PR37Y15 maize rhizosphere (Supplementary Figure S4). Moreover, the pseudomonads proportions of *phlD*^+^, *prnD*^+^, *pltC*^+^, and *phzCD*^+^ were higher in PR37Y15 maize rhizosphere than in DK315 maize rhizosphere in the two soils Bmo1 and Ysa8. Similarly, a plant selection effect was found toward *phlD*^+^ pseudomonads isolates between three different maize genotypes, showing a significant higher abundance of *phlD*^+^ pseudomonads in the rhizosphere of a maize hybrid than in its parent lines (Picard and Bosco, 2005). Indeed, maize genotypes may select distinct plant-beneficial functional groups. In turn, these plant genotypes may respond differentially to PGPR (Walker et al., 2011), which can express plant-beneficial genes at different levels according to maize genotype (Rice et al., 2011; Vacheron et al., 2016).

Among the seven subgroups (Mulet et al., 2010) evidenced here from the *P. fluorescens* group, the four FMJK subgroups included pseudomonads with a lower number of co-occurring plant-beneficial properties compared with the three CPC subgroups, in which biocontrol properties were more prevalent. It is interesting to note that the lower rhizosphere competitiveness of CPC pseudomonads tends to affect particularly strains with 6–9 plant-beneficial properties in comparison with CPC pseudomonads with 1–5 of these properties (**Figure 6B**). Results suggest that there is a trade-off between rhizosphere prevalence and the ability to maintain a large number of plant-beneficial properties in the rhizosphere environment, where competition for rhizodeposits and other root exudates is high (Bais et al., 2006). This may have functional implications, as the two CPC and FMJK *Pseudomonas* subgroups tend to implement different arrays of plant-beneficial effects (phytostimulation vs. biocontrol) in the rhizosphere, and it is tempting to speculate that their joint contributions may be useful to optimize symbiotic benefits for root system functioning. Even if maize selected *Pseudomonas* rhizobacteria harboring few plant-beneficial properties (<5), all plant-beneficial properties were represented in the rhizosphere *Pseudomonas* populations. Maize may maximize the taxonomic diversity and functional redundancy of *Pseudomonas* populations, thereby allowing a better allocation of ecological tasks among the rhizomicrobiota. This is in line with co-inoculation experiments of different bacterial and/or fungal strains, which offer a functional complementarity between the plant-beneficial actions they display and enhanced positive effects on the host plant (Srivastava et al., 2010; Combes-Meynet et al., 2011; Couillerot et al., 2012). Indeed, the inoculation of consortia may also favor other symbioses in the rhizosphere, as the inoculation of two pseudomonads increased the number of rhizobial root nodules and improved symbiotic performance (Egamberdieva et al., 2010).

## Conclusion

This report suggests that plants shape the composition of *Pseudomonas* populations by preferentially selecting pseudomonads harboring one to five plant-beneficial properties. This is the first study that characterizes fluorescent *Pseudomonas* populations in the rhizosphere according to the type and number of plant-beneficial properties they harbor and their taxonomic status.

## Author Contributions

JV, DM, and CP-C conceived and designed the experiments. JV performed the experiment. MG-M contributed to plant hormone analysis. JV, YM-L, DM, AD, and CP-C analyzed the data and JV, YM-L, DM, and CP-C wrote the manuscript; all authors contributed to the discussion and approved the final manuscript.

## Conflict of Interest Statement

The authors declare that the research was conducted in the absence of any commercial or financial relationships that could be construed as a potential conflict of interest.
