# Supplementary material for: Fluorescent Pseudomonas Strains with only Few Plant-Beneficial Properties Are Favored in the Maize Rhizosphere
Source: Front Plant Sci. 2016 Aug 25;7:1212. doi: 10.3389/fpls.2016.01212 (PMC4996994; doi:10.3389/fpls.2016.01212)
Supplement: Supplementary file 1 [file Presentation_1.PDF]

Supplementary data

Vacheron *et al.*

Frontiers in Plant Science

Table S1: Physical and chemical characteristics of soils used in this study

Characteristics	Soil MS8	Soil Ysa5	Soil Ysa8	Soil Bmo1
Location	Morens (Switzerland)	Seyssel (France)	Seyssel (France)	Béligneux (France)
Vegetative cover	Artificial meadow	Maize	Grassland	Maize
Land use	Maize for at least 2 years	Maize for at least 2 years	Never cultivated	Maize for at least 2 years
Geologic origin	Morainic	Sandstone	Sandstone	Morainic
Textural class	Sandy loam	Sandy loam	Loamy sand	Loamy sand
Clay (%)	10.7	17.8	18.5	11.5
Loam (%)	29.6	25.9	25.5	26.2
Sand (%)	59.7	56.3	55.9	62.4
pH (water)	7.8	7.9	7.9	6.6
Organic matter (%)	1.3	2.2	3.8	2.4
$CaCO_3$ total (%)	6.0	2.8	5.8	1.4
N total (%)	0.13	0.16	0.17	0.17
P total (mg.kg $^{-1}$)	750	382	54	322
K total (mg.kg $^{-1}$)	150	223	136	158
Ca total (g.kg $^{-1}$)	8.47	6.86	8.05	2.14
Mg total (mg.kg $^{-1}$)	103	184	231	72
Fe [$C_2H_4O_2$ 1:10] (mg.kg $^{-1}$)	230	32.2	141	ND

Table S2: List of primers used in this study

Traits	Targeted genes	Primers	Sequences [a]	References
DAPG production	*phlD*	B2BF	ACC CAC CGC AGC ATC GTT TAT GAG C	McSpadden Gardener *et al.* 2001
		BPR4	CCG CCG GTA TGG AAG ATG AAA AAG TC	
ACC deaminase activity	*acdS*	accF5	GGC AAC AAG MYS CGC AAG CT	Bouffaud, personal communication
		accR8	CTG CAC SAG SAC GCA CTT CA	
Nitrogen fixation	*nifH*	PolF	TGC GAY CCS AAR GCB GAC TC	Poly *et al.* 2001
		PolR	ATS GCC ATC ATY TCR CCG GA	
NO production	*nirS*	cd3a	GTS AAC GTS AAG GAR ACS GG	Throbäck *et al.* 2004
		R3cd1322	GAS TTC GGR TGS GTC TTG A	
Pyrrolnitrin production	*prnD*	prnD1	GGG GCG GGC CGT GGT GAT GGA	de Souza *et al.* 2003
		prnD2	YCC CGC SGC CTG YCT GGT CTG	
Pyoluteorin production	*pltC*	pltC1	AAC AGA TCG CCC CGG TAC AGA ACG	de Souza *et al.* 2003
		pltC2	AGG CCC GGA CAC TCA AGA AAC TCG	
Phenazin production	*phzC-phzD*	PHZ1	AAC AGA TCG CCC CGG TAC AGA ACG	de Souza *et al.* 2003
		PHZ2	AGG CCC GGA CAC TCA AGA AAC TCG	
Sigma 70	*rpoD*	rpodf	ACT TCC CTG GCA CGG TTG ACC A	Frapolli *et al.* 2007
		rpodr	TCG ACA TGC GAC GGT TGA TGT C	
16S ribosomal RNA	*rrs*	pA	AGA GTT TGA TCC TGG CTC AG	Edwards *et al.* 1989
		pH	AAG GAG GTG ATC CAG CCG CA	

[a] IUPAC convention used for the description of degenerate DNA sequences : Y = C/T; S = G/C; R = A/G.

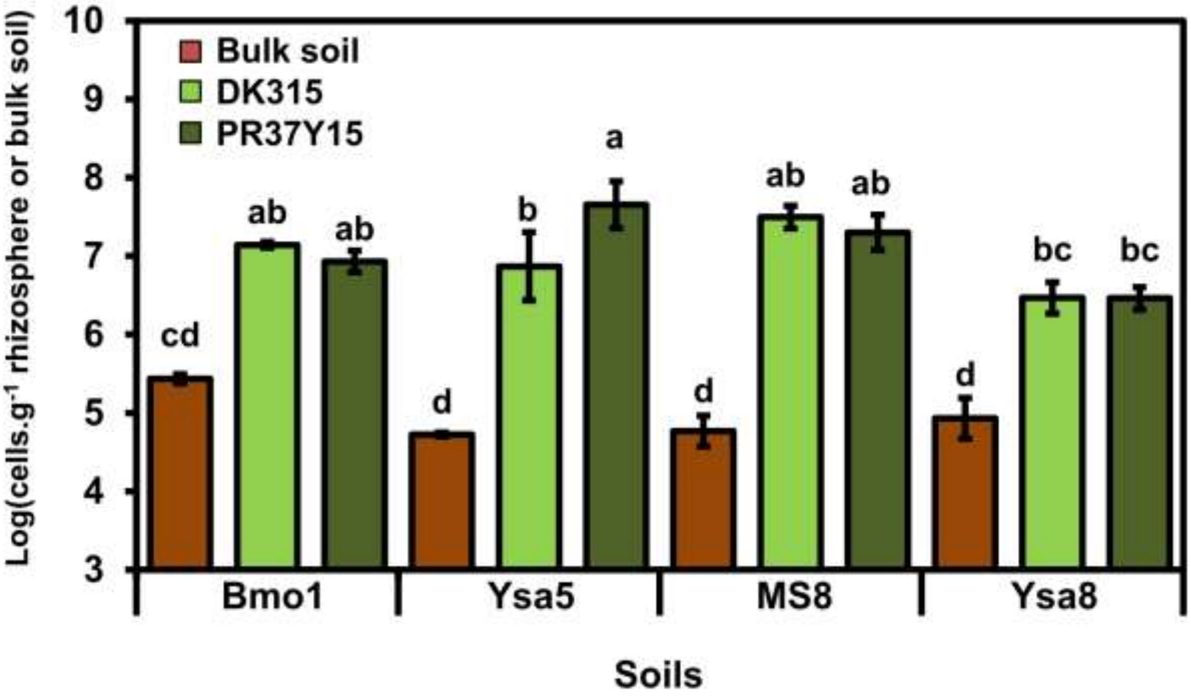

Figure S1: Abundance of culturable fluorescent *Pseudomonas* in bulk soil and rhizosphere of maize cultivars DK315 and PR37Y15 at 21 days in soils Bmo1, Ysa5, MS8 and Ysa8. Error bars correspond to standard errors (n=4). For each soil, letters a-d indicates statistical relations (ANOVA and Tukey's HSD tests, $P < 0.05$) between conditions (bulk soil/PR37Y15/DK315).

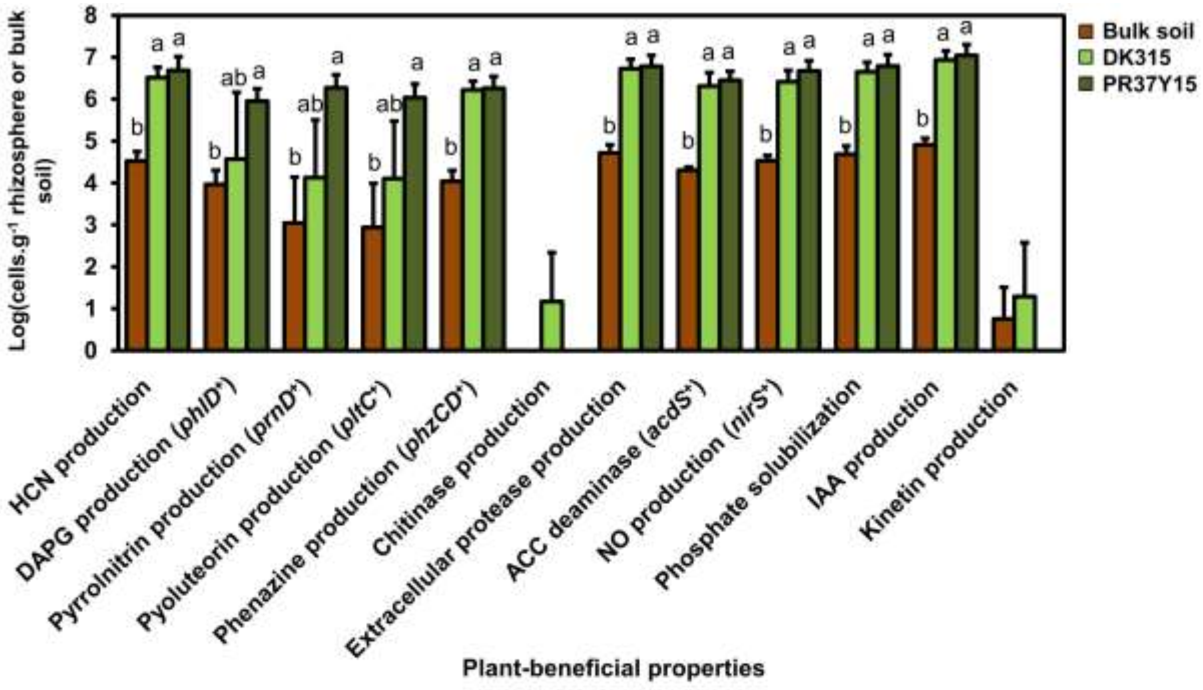

Figure S2: Estimation of the number of pseudomonads according to the type of plant-beneficial properties harbored, in bulk soil and in the rhizosphere of maize cultivars DK315 and PR37Y15. Data from soils Bmo1, Ysa5, MS8 and Ysa8 were combined soils. The number of *Pseudomonas* was estimated according to the extrapolation of proportions to the results of *Pseudomonas* enumeration for each condition. HCN: Hydrogen cyanide; DAPG: 2,4-diacetylphloroglucinol; ACC: 1-amino-cyclopropane carboxylic acid; NO: Nitric oxide. Error bars correspond to standard error. Statistical differences between conditions (bulk soil/PR37Y15/DK315) are indicated with letter a-b (ANOVA, Fisher's LSD tests, $P < 0.05$).

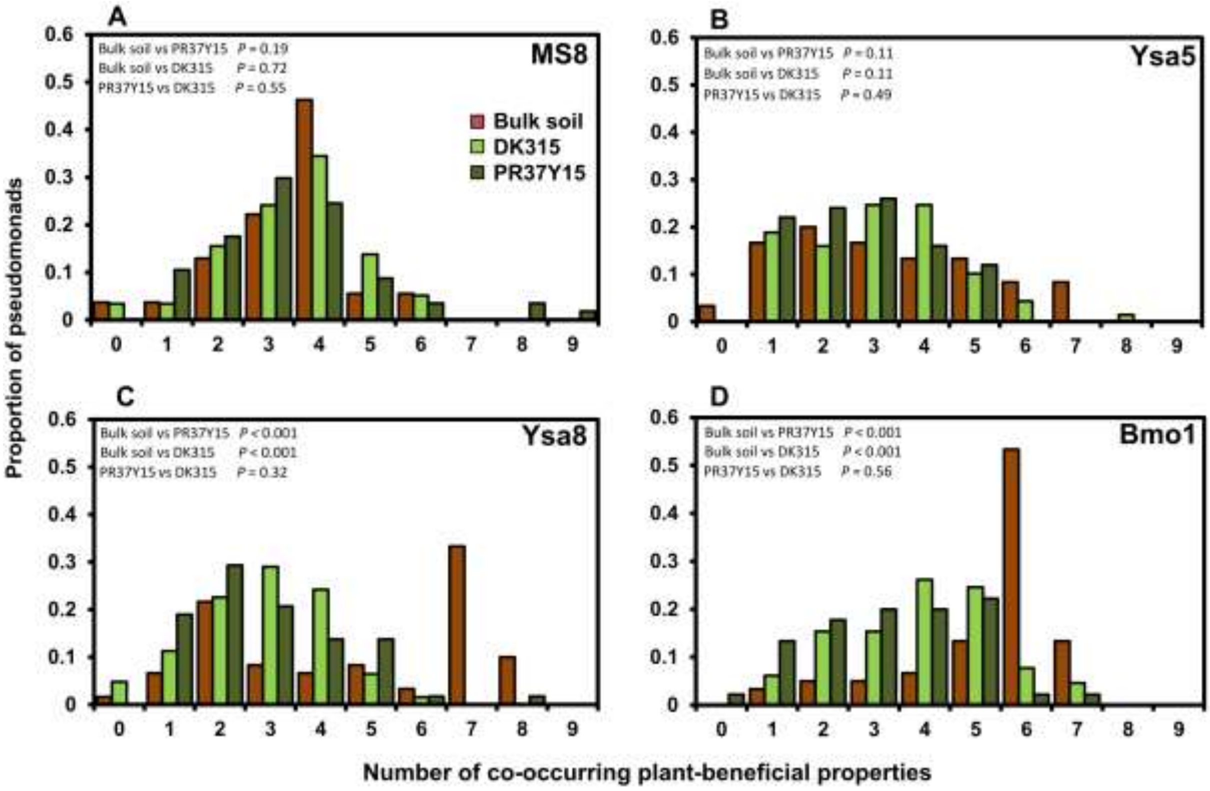

Figure S3: Distribution of the proportion fluorescent *Pseudomonas* according to the number of co-occurring plant-beneficial properties harbored (from 0 to 9), in bulk soil and the rhizosphere of maize cultivars DK315 and PR37Y15 at 21 days in soils MS8 (a), Ysa5 (b), Ysa8 (c) and Bmo1 (d). The *P* values are shown when distributions differed according to χ^2 tests.

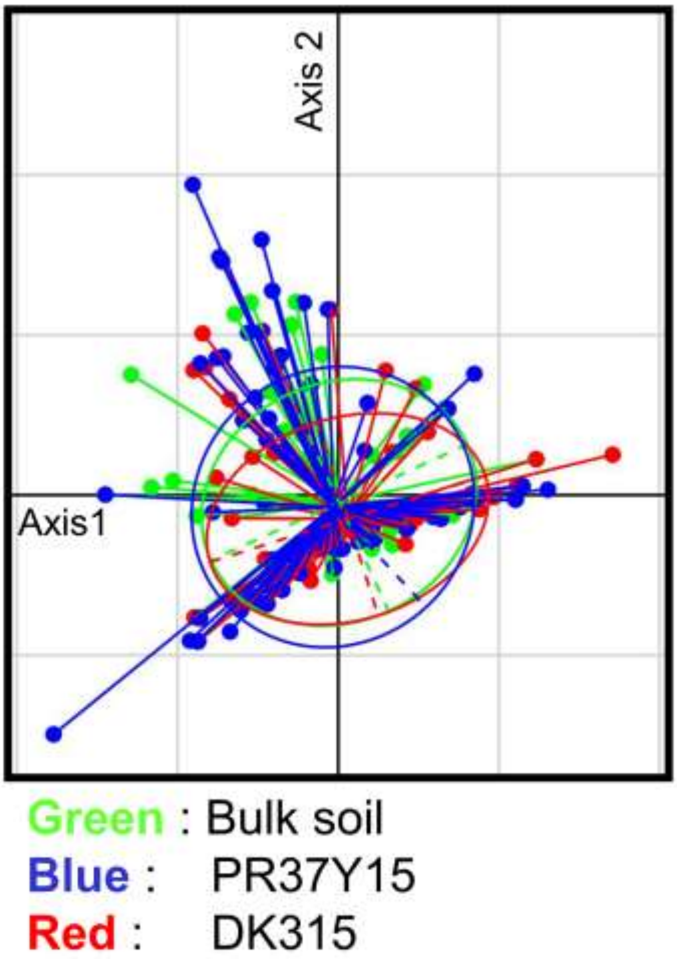

Figure S4: Factorial correspondence analysis (FCA) showing the repartition of the 698 functional profiles of *Pseudomonas* isolates according to conditions (bulk soil, rhizospheres PR37Y15, and DK315). Axis 1 and axis 2 correspond to respectively 20.6 % and 19.1% of the total variance. Each point of the FCA corresponds to the profile of one isolate. Circles correspond to confidence ellipses.

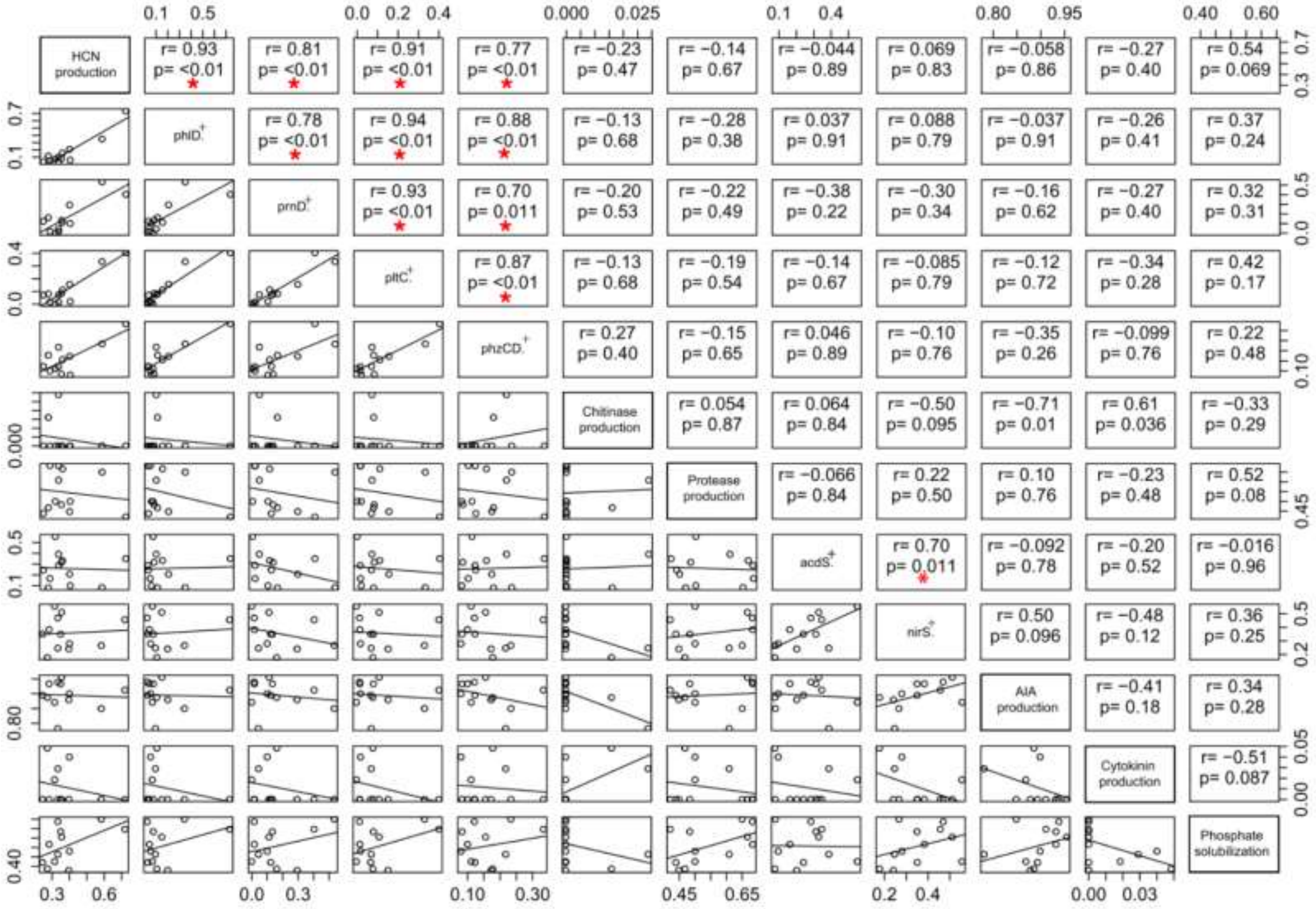

Figure S5: Co-occurrence of plant-beneficial properties harbored by pseudomonads. For each comparison, Pearson correlation coefficient r and *P* value (with a red star when $P < 0.05$) are indicated.

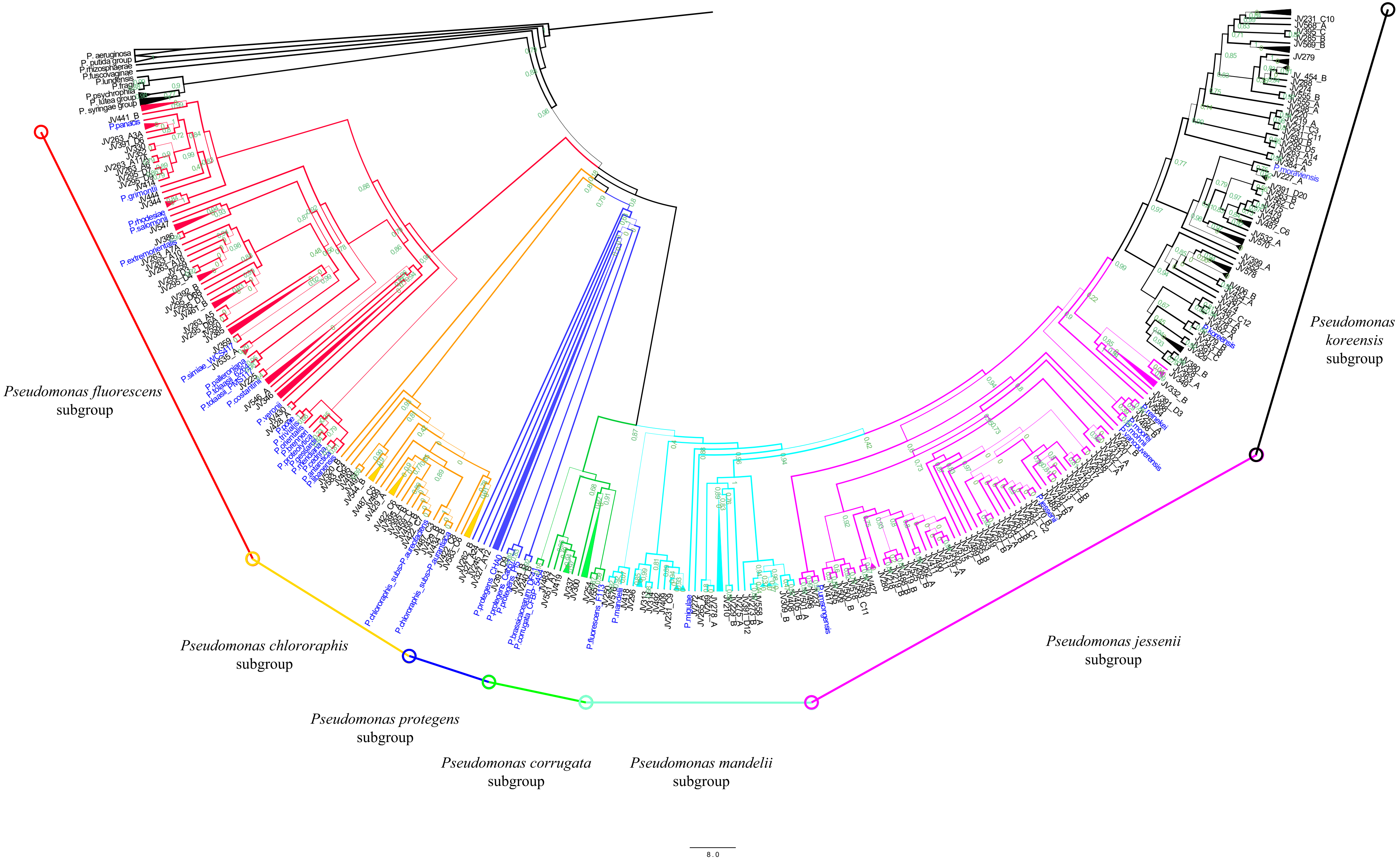

Pseudomonas fluorescens subgroup
Pseudomonas chlororaphis subgroup
Pseudomonas protegens subgroup
Pseudomonas corrugata subgroup
Pseudomonas mandelii subgroup
Pseudomonas jessenii subgroup
Pseudomonas koreensis subgroup

Figure S6: Phylogenetic tree of 498 isolates taxonomically characterized based on their *rpoD* sequence.

Phylogenetic analysis of the *rpoD* housekeeping gene was based on 30 type strains (Written in blue, Mulet *et al.* 2010), 9 non-type strains and a clade of 7 type strains from the *P. aeruginosa* group (used for tree rooting). Other *Pseudomonas* groups such as the *P. syringae*, *P. putida* and *P. lutea* groups, as well as non-clustered *Pseudomonas* strains are represented. The maximum likelihood tree was inferred using PhyML and the GTR model, and nodal robustness was assessed using 500 bootstrap replicates. Red: *P. fluorescens* subgroup; Orange: *P. chlororaphis* subgroup; Blue: *P. protegens* subgroup; Green: *P. corrugata* subgroup; Light blue: *P. mandelii* subgroup; Pink: *P. jesseni* subgroup; Black: *P. koreensis* subgroup.

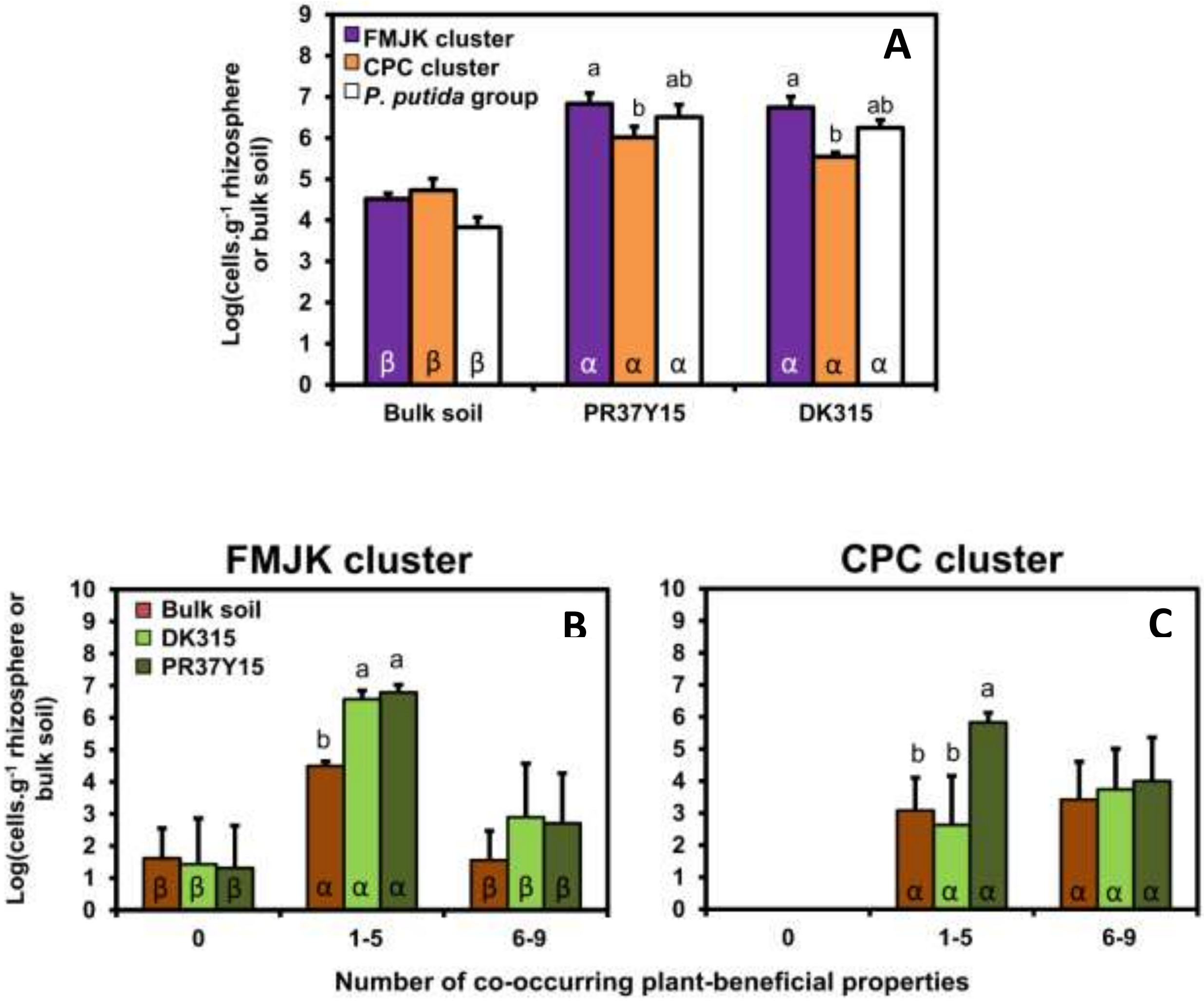

Figure S7: Comparison of the estimated number of pseudomonads belonging to FMJK and CPC clusters and to *P. putida* group in bulk soil, and in the PR37Y15 and DK315 rhizospheres (A) and according to the number of co-occurring plant-beneficial properties harbored (B and C). Error bars correspond to standard errors. Statistical differences between subgroups are indicated with letter a-b and statistical differences between conditions (bulk soil/PR37Y15/DK315) are indicated with letter α-β (ANOVA, Fisher's LSD tests, $P < 0.05$).
